# Synthesis and Raman Detection of 5-Amino-2-mercaptobenzimidazole Self-Assembled Monolayers in Nanoparticle-on-a-Mirror Plasmonic Cavity Driven by Dielectric Waveguides

**DOI:** 10.1021/acs.nanolett.3c04932

**Published:** 2024-03-14

**Authors:** Javier Redolat, María Camarena-Pérez, Amadeu Griol, Miguel Sinusia Lozano, Maria Isabel Gómez-Gómez, J. Enrique Vázquez-Lozano, Ermanno Miele, Jeremy J. Baumberg, Alejandro Martínez, Elena Pinilla-Cienfuegos

**Affiliations:** 1 Nanophotonics Technology Center, Universitat Politècnica de València, Valencia E46022, Spain; 2 Department of Electrical, Electronic and Communications Engineering, Institute of Smart Cities (ISC), Universidad Pú́blica de Navarra (UPNA), 31006 Pamplona, Spain; 3 NanoPhotonics Centre, Cavendish Laboratory, Department of Physics, University of Cambridge, Cambridge CB3 0HE, United Kingdom

**Keywords:** interface engineering, self-assembled monolayers, gold surfaces, nanoparticle assembly, chemical functionalization, nanoparticle on a mirror cavity, integrated waveguides, on-chip spectroscopy, molecular optomechanics

## Abstract

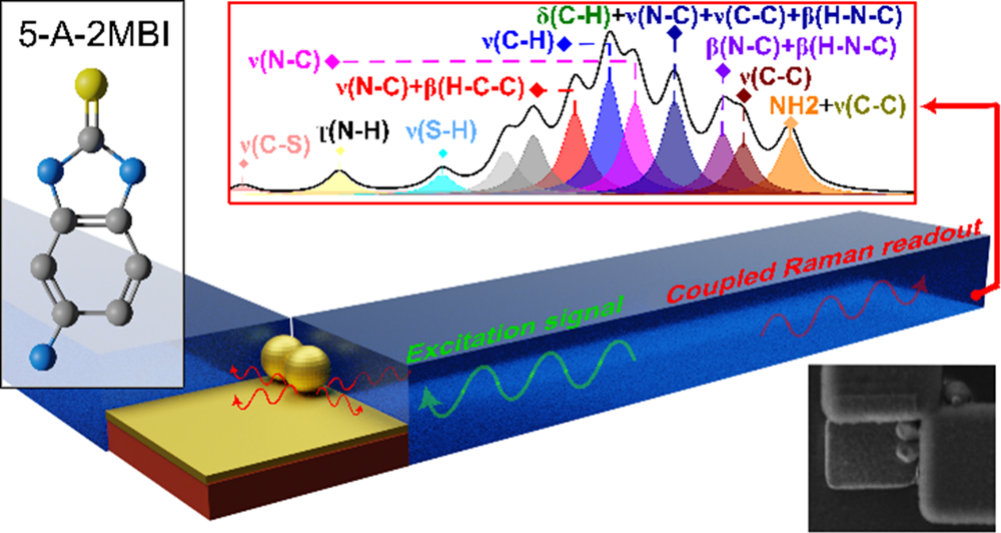

Functionalization of metallic surfaces by molecular monolayers is a key process in fields such as nanophotonics or biotechnology. To strongly enhance light–matter interaction in such monolayers, nanoparticle-on-a-mirror (NPoM) cavities can be formed by placing metal nanoparticles on such chemically functionalized metallic monolayers. In this work, we present a novel functionalization process of gold surfaces using 5-amino-2-mercaptobenzimidazole (5-A-2MBI) molecules, which can be used for upconversion from THz to visible frequencies. The synthesized surfaces and NPoM cavities are characterized by Raman spectroscopy, atomic force microscopy (AFM), and advancing–receding contact angle measurements. Moreover, we show that NPoM cavities can be efficiently integrated on a silicon-based photonic chip performing pump injection and Raman-signal extraction via silicon nitride waveguides. Our results open the way for the use of 5-A-2MBI monolayers in different applications, showing that NPoM cavities can be effectively integrated with photonic waveguides, enabling on-chip enhanced Raman spectroscopy or detection of infrared and THz radiation.

Recent years have witnessed a growing interest in understanding how the arrangement of atoms and molecules and their interactions at the interfaces of complex systems influence the behavior and capabilities of multifunctional devices. These interfaces play a crucial role in shaping the functionality and performance of these devices, which has led to significant interest in different fields of research. Therefore, understanding and manipulating interfaces at the nanoscale hold the key to unlocking new possibilities in fields such as catalysis,^1^ sensing,^2^ electronics,^3^ and biotechnology.^4^ In this context, the functionalization of surfaces with tailored molecular layers has become a fundamental approach to control and optimize interface properties.^5^

Within this framework, gold (Au) surfaces have garnered significant attention due to their unique chemical and physical properties. Functionalizing Au surfaces, either flat or in the form of nanoparticles (NPs), with self-assembled monolayers (SAMs) has proven to be an effective strategy for tailoring surface chemistry and introducing new functionalities.^6^ SAMs, formed through the spontaneous adsorption of organic molecules onto a substrate, provide a versatile platform for surface modification, enabling precise control over surface properties such as wettability, charge, and reactivity.^7^ Moreover, by controlling the sizes and shapes of Au-NPs, it becomes possible to further fine-tune the optical properties that arise from the reduction in dimensionality. This includes the ability to manipulate plasmonic resonances,^8^ which play a crucial role in enhancing light–matter interactions and hold great potential for applications in fields such as sensing, spectroscopy, and optical devices.^9,10^ Therefore, localized plasmonic resonances in metallic nanoparticles have been engineered for reaching spectroscopic fingerprinting down to the single molecule level, enabled by the extraordinary enhancement of Raman scattering in the near field of metallic NPs, surfaces (SERS),^11^ or tips (TERS).^12^

A well-known example is the nanoparticle on a mirror (NPoM) cavity, which has been used to achieve extreme light confinement within pico- and nanocavities, allowing atomic-scale spectroscopy of individual molecules via SERS.^13,14^ In this type of nanocavity, a plasmonic NP is separated from an underlying metal film (mirror) by a molecular monolayer. To fabricate such nanocavities, bottom-up nano assembly of metallic surfaces and plasmonic NP immobilization are required.^15^ The space between the NP and the mirror bridged by bonding molecules displays a strong electromagnetic field enhancement below the diffraction limit at resonance, which allows efficient SERS^13^ and, in general, improves any effect relying on light–matter interaction. Remarkably, combining molecular optomechanics with the extreme field localization in the gap of NPoM cavities enables frequency upconversion from the mid-infrared (MIR) to the visible domain at ambient conditions by parametrically coupling the molecular vibrations of the monolayer to the optical resonance of a plasmonic antenna.^16,17^ In this scheme, the detected frequency corresponds to the vibrational state of the molecule placed in the NPoM gap. Therefore, upconversion-based detection of other infrared (IR) or even terahertz (THz) frequencies depends on our capacity to functionalize Au surfaces with a wide variety of molecular monolayers that should provide a large Raman scattering cross-section and a high IR absorption. Amongst the different molecules providing both requirements (to be Raman and IR active), 5-amino-2-mercaptobenzimidazole (5-A-2MBI)^18^ exhibits this property in the THz range (15–60 THz).^19^

In this work, we present a new strategy for effective functionalization of Au surfaces with 5-A-2MBI SAMs.^20,21^ We investigate the synthesis and characterization of the 5-A2MBI SAM and its homogeneity, stability, and surface coverage. This involves the SAM strongly bonding to Au flat surfaces through a thiol covalent-metallic interaction. Changes in the wetting behavior of the Au surface performed before and after the SAM synthesis by advancing and receding water contact angle (WCA) measurements confirmed the presence of the SAM. Its structure, in terms of homogeneity, flatness, and lack of clusters or islands, was characterized by atomic force microscopy (AFM) topography images. Additionally, by exploiting the affinity of the NH_2_ functional group, citrate-capped Au-NPs were assembled on the 5-A-2MBI surfaces to form NPoM cavities. Furthermore, we show that the affinity between 5-A-2MBI and Au-NPs can be enhanced by protonating the 5-A-2MBI SAM, and therefore, the Au-NP deposition onto the functionalized Au-5-A-2MBI surfaces can be increased, enabling control over the assembly of Au-NPs. Finally, the formation of the 5-A-2MBI SAM was confirmed by means of SERS detection in NPoM structures by Raman spectroscopy, which allowed the acquisition of the Raman fingerprint. As a step forward, on-chip Raman detection in NPoM cavities driven by silicon nitride waveguides is also performed. This NPoM-on-chip integration, mixing standard lithography with an advanced NP transfer method,^15^ opens the door to advanced Raman spectroscopy and, ultimately, molecular-optomechanics THz detection on silicon-based photonic integrated circuits.

Figure 1 shows a sketch of the 5-A-2MBI molecule.^18^ The IR, Raman, and upconversion response of 5-A-2MBI molecule extracted from the online platform *Molecular Vibration Explorer*(19) are shown in Figure 1b. With this tool, we were able to simultaneously theoretically investigate IR absorption, Raman scattering, and vibrational sum and difference frequency generation cross sections. Moreover, it allows the selection of polarization vectors for the electromagnetic fields to set molecular orientations and customize parameters for plotting corresponding IR, Raman, and sum-frequency spectra in the THz range.

**Figure 1:**
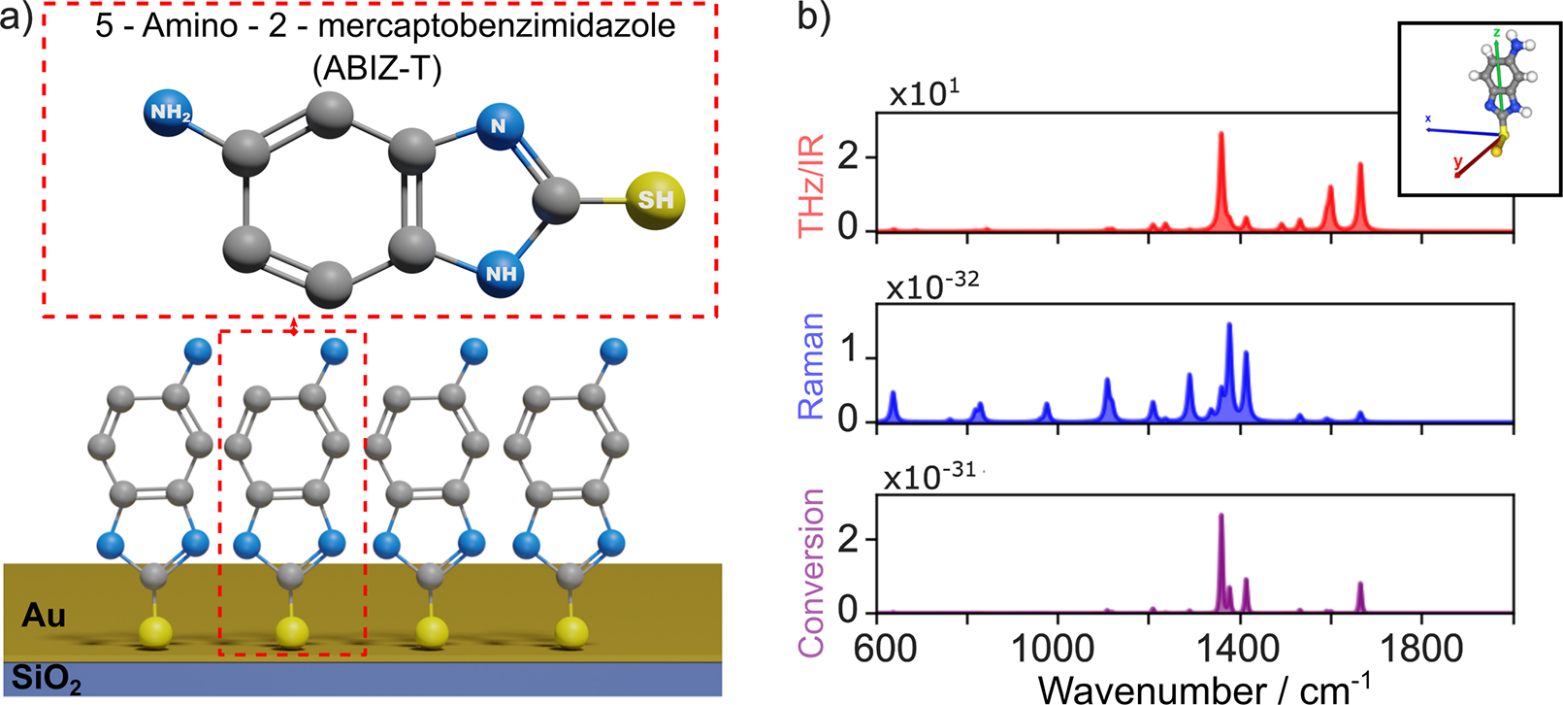
5-A-2MBI SAM formation and simulated response. a) 5-A-2MBI molecule and schematic SAM structure formed on a Au surface. b) IR, Raman and up-conversion spectra obtained by simulations at the range of 15–60 THz (500–2000 cm^–1^), considering a 532 nm Raman laser, *x*-polarization, ambient conditions of 5-A-2MBI molecule from ref (19). Inset: 5-A-2MBI molecule orientation. SMILES code: Nc1ccc2c(c1)[nH]c(n2)S. Note on spectra units: THz/IR [km·mol^–1^], Raman [cm^2^·sr^–1^], conversion [km·mol^–1^·cm^2^·sr^–1^].

For our experiments, we obtained commercial 5-A-2MBI (99%) from Merck-Sigma Aldrich. Its empirical formula is C_7_H_7_N_3_S, and its molecular weight is 165.22 g/mol.^18^ In the functionalization process, first a piranha solution (H_2_SO_4_:H_2_O_2_, 1:1) was used for beaker cleaning. The Au substrates were previously cleaned with isopropanol solution, thoroughly rinsed with absolute ethanol, and finally dried with N_2_. 5-A-2MBI SAMs were then prepared by dipping the substrates in a 10 mM 5-A-2MBI in ethanol solution (absolute, reagent grade) for 16 hours. Finally, the sample was sonicated in ethanol for 3 min, rinsed with ethanol, and dried under N_2_ stream. Different WCA values were obtained for three cases, probing functionalization with the 5-A-2MBI SAM on Au surfaces: 76° for non-functionalized gold; 62° for non-functionalized gold + ethanol; 54° for functionalized gold (see details in Supporting Information section SI.1). AFM measurements were performed, and uniform surfaces without clusters or islands were obtained (see details in Supporting Information section SI.2).

To improve the Au-NP deposition onto the functionalized Au-5-A-2MBI surfaces, the affinity between 5-A-2MBI molecules and Au-NPs can be enhanced by protonating the 5-A-2MBI SAM immersing the samples in an acidic solution.^22^ Comparing the results before and after protonation of the 5-A-2MBI SAM, a remarkable increase of approximately 800% in the density of 60 nm Au-NPs is evident, as well as an increase of approximately 300% in the density of 150 nm Au-NPs (see details in Supporting Information section SI.3).

Raman spectroscopy characterization was performed on the Au-5-A-2MBI SAM functionalized samples, with additional information about the measurement in Supporting Information section SI.4. In particular, SERS measurements were performed on NPoM cavities prepared by 60 nm Au-NP drop casting on

5-A-2MBI functionalized Au flat samples (Figure 2a) were used to enhance the 5-A-2MBI molecule Raman response. Figure 2c shows a Raman image that was generated from changes in the Raman peak at 1586 cm^–1^ assigned to the amine stretching vibrations. An area of 20 × 20 μm^2^ was scanned, and as it can be seen in the figure, bright spots correspond to the SERS response of several Au-NPs dispersed on the Au-5-A-2MBI functionalized surface forming several NPoM cavities. Figure 2b depicts the experimental Raman spectrum obtained at the Au-NP center (highlighted in the Raman image) in comparison with the experimental Raman spectrum obtained for the bulk sample (molecules in powder). The experimental Raman spectra were then fitted using a Lorentzian function, and some of the most representative Raman peaks of the 5-A-2MBI molecule have been labeled with numbers corresponding to the vibrational modes of the molecule detailed in Table 1.

**Figure 2:**
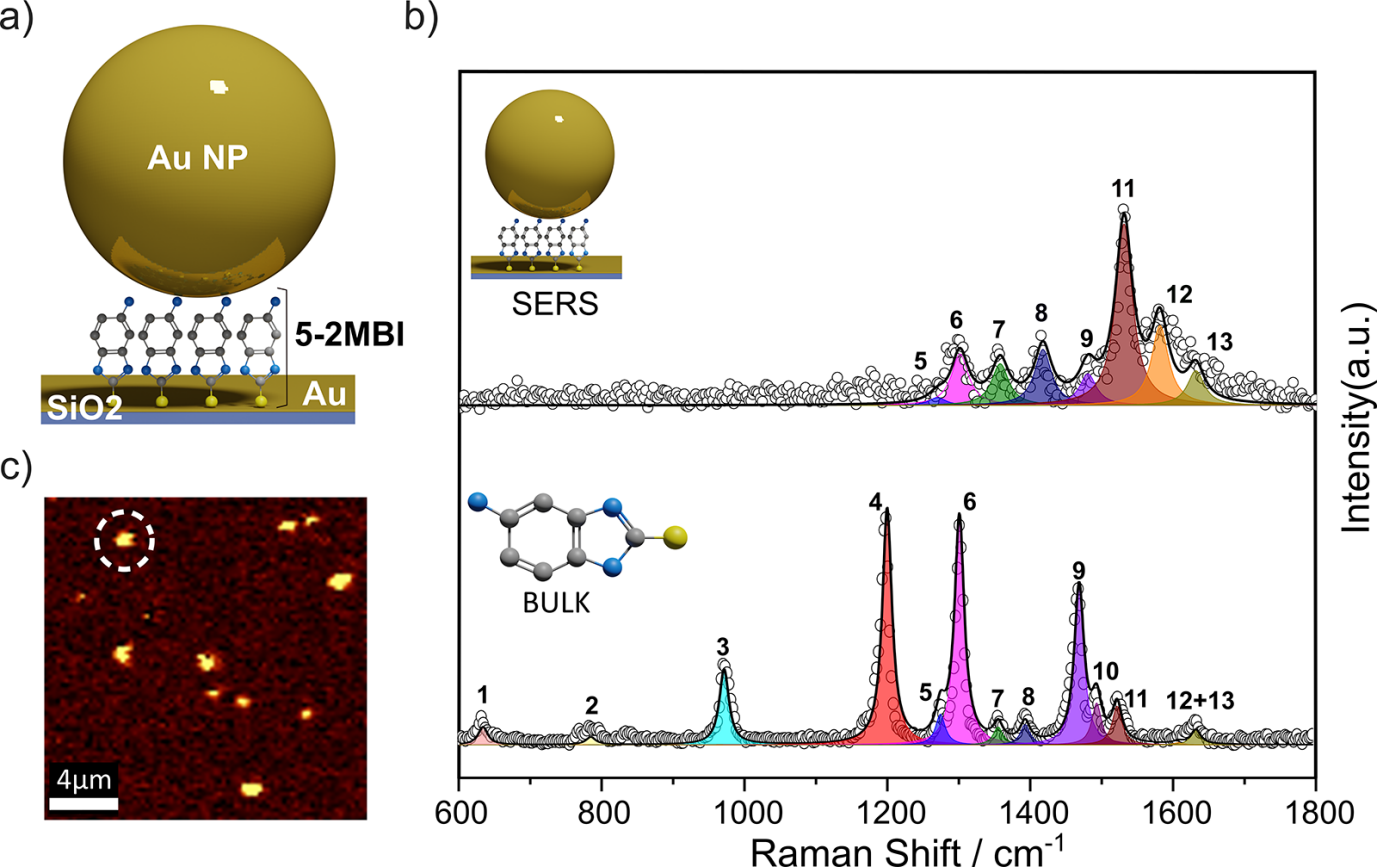
a) Schematic representation of the NpoM structure. b) Top curve: Experimental SERS spectrum at the center of the highlighted Au-NP in panel b) (dotted plot) along with Lorentzian peak fitting (shaded colours). Bottom curve: Experimental Raman spectrum of the 5-A-2MBI molecule (powder) (dotted plot) with Lorentzian peak fitting (shaded colours). Raman peaks are numerically labelled, and corresponding vibrational modes are detailed in Table 1. c) Raman image depicting the intensity of the 1586 cm^–1^ Raman peak, corresponding to amine stretching vibrations (Scan: 20 × 20 μm^2^).

**Table 1:** Comparison of the Calculated Vibrational Frequencies (cm^–1^)^19^ and SERS Vibrational Frequencies (cm^–1^) of the Experimental Results of This Work for BULK, Single NPoM, and Guided NPoM Configuration^a^

Peak	ν (cm^–1^)^19^	ν (cm^–1^) [BULK]	ν (cm^–1^) [NPoM]	ν (cm^–1^) [Guided]	Vibrational modes
1	638	633		626	ν(C–S)
2	830	784		792	τ(N–H)
3	976	975		973	ν(S–H)
4	1209	1201		1197	ν(N–C) + β(H–C–C)
5	1289	1278	1286	1265	ν(C–H)
6	1338	1304	1301	1305	ν(N–C)
7	1358	1359	1355	1391	δ(C–H)
8	1376	1395	1408		ν(N–C) + ν(C–C) + β(H–N–C)
9	1414	1464	1479	1457	β(N–C) + β(H–N–C)
10		1495			ν(C–C) + ν(N–C) + β(H–C–C)
11	1531	1525	1528	1530	ν(C–C)
12	1587	1610	1586	1580	Broad scissor primary −NH_2_
13	1665	1636	1632		ν(C–C)

a ν, stretching; δ, β, in-plane bending; τ, out-of plane bending.

Certain applications may benefit from having the target functionality implemented in a photonic silicon integrated circuit. In the specific cases of Raman spectroscopy or THz detection, the advantages of photonic integration would include low-cost fabrication at large volumes, portability, or the possibility of multiplexing multiple detection channels on a single chip, among many others. Raman detection after molecule excitation using the evanescent field of guided modes in long silicon nitride waveguides has been already reported in the literature.^23^ To reduce the total foot-print, plasmonic cavities such as bow-tie nanoantennas can be built either on top of the waveguide^24^ or in a waveguide gap to ensure highly efficient illumination and detection.^25^ However, the plasmonic gap in bow-tie nanoantennas is defined by lithography, making it extremely challenging to attain the subnanometer spacing that is exploited in NPoM cavities. Recently, it has been shown numerically that silicon nitride waveguides can be used to illuminate molecules centred in the gap of an NPoM as well as to collect efficiently the Raman signal scattered by them,^26^ but to the best of the authors knowledge, there has not been any experimental demonstration. Here, we have explored this approach in practice to experimentally measure the Raman spectrum of a Au-5-A-2MBI SAM.

Essentially, the waveguide-driven NPoM approach consists of a hybrid plasmonic-photonic system where a square Au patch (the mirror of the NPoM cavity) is lithographically defined at the intersection of two strip SiN waveguides, forming an in-plane L-shaped arrangement (see Figure 3a). Therefore, the NPoM cavity is physically situated in the corner linking two orthogonal waveguides so the molecules in the plasmonic gap can be in-plane pumped by any of the two waveguides. The generated Raman signal can be collected by the same waveguide (in reflection mode, input/output 1 in Figure 3a) or by the perpendicular one (transmission mode, input/output 2 in Figure 3a) using the transverse magnetic (TM) guided mode for both cases to ensure that the electric field is perpendicular to the gap. In the transmission mode, the Raman noise generated by the pump when propagating through the input waveguide^27^ can be significantly decreased at the output. In this intersecting area, the metallic Au patch is functionalized with the 2-A-5MBI SAM. Ideally, by using a soft-lithography method for deterministic positioning of NPs,^15^ one single NP could be positioned on top of the Au patch (more details in Supporting Information section SI.5). Nevertheless, in our experiments, we obtained two 150 nm Au-NPs on the patch and in contact with one of the waveguide facets after the positioning process, and this situation is shown in the sketch of Figure 3a (see also Figure 4). Although a higher intensity enhancement factor results when positioning the NP at the center of the Au mirror patch, the enhancement factor peak for NPs placed at the borders of the waveguides still retains the same order of magnitude. Indeed, we simulated numerically the electromagnetic field enhancement in the NPoM gap as well as the collection efficiency (β factor) for both NPs.^26^ The simulations were performed with CST Microwave studio for two orthogonal silicon nitride waveguides (650 × 220 nm^2^ cross-section for each one), with a thin Au patch (650 × 650 nm^2^ sides and 20 nm height) placed at the intersection of the waveguides. Also, we considered a 150 nm diameter gold NP positioned on a specific zone (NP1 and NP2 in Figure 3a) following the positioning obtained in the real experiment.

**Figure 3:**
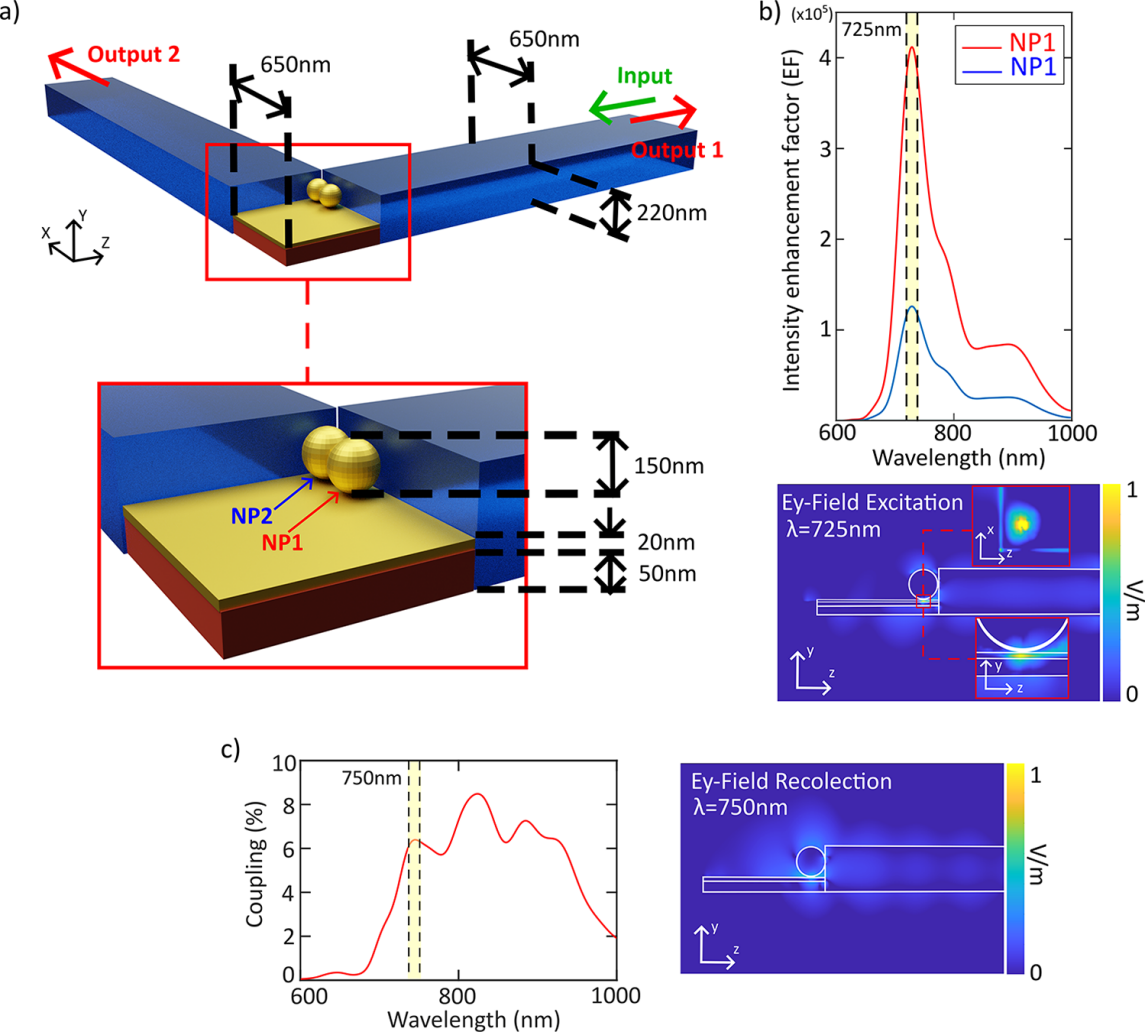
a) Schematic view of the NPoM configuration at the intersection of two perpendicular silicon nitride waveguides. Inset: detail of the NPoM cavity. b) Simulation of the excitation efficiency: enhancement intensity factor (EF) in the gap below both NPs when illuminated using the TM waveguide mode (top panel); snapshot of the electric field when injecting the TM mode at 725 nm, including insets showing the hot-spot arising from exciting the (1, 0) mode of the NPoM. c) Simulation of the collection efficiency: calculation of the β-factor spectrum (left panel) and snapshot of the vertical electric field when placing dipole at a 750 nm wavelength in the NPoM gap.

**Figure 4:**
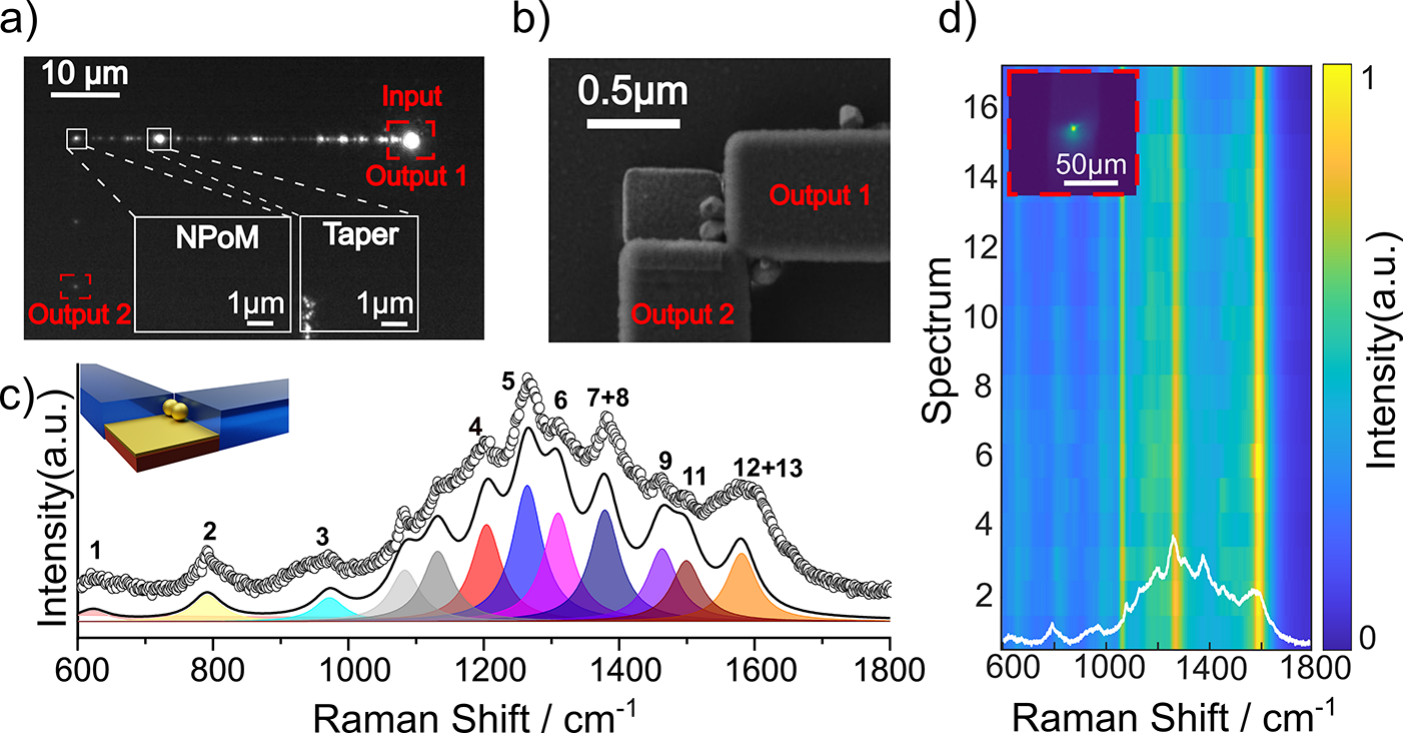
a) Upscattered image of the light coupling into the waveguide and traveling through it exciting the on-chip NPoM. Also seen are two bright spots which are the NPoMs (left) and taper (right). Collected Raman light emerging from waveguide is seen on upper right. b) SEM image of transferred NPs to create NPoM configuration integrated with orthogonal waveguides. c) Experimental Raman spectrum (dotted curve) with Lorentzian peak fitting (shaded colours). Raman peaks are numerically labeled, and corresponding vibrational modes are detailed in Table 1. d) Raman spectra tracked during successive measurements.

The enhancement of the electromagnetic field confinement in the SAM was evaluated using a probe field below the NP, just in the middle of the monolayer (which was modelled assuming a thickness of 1 nm and a refractive index of 1.8). We calculated the intensity enhancement factor as EF(*r*, λ) ≡ |*E*(*r*, λ)|^2^/|*E*_0_(*r*, λ)|^2^, where *E*(*r*, λ) is the calculated electric field at the SAM below the NP with and *E*_0_(*r*, λ) is the field in the same position when the metallic patch and the NP are removed. Simulation details are given in Supporting Information section SI.6. In the top panel of Figure 3b, we can see that the electric field in the SAM is extremely enhanced (above 10^5^) around the NPoM resonance wavelength (725 nm) and within a wavelength range that spans from 650 to 800 nm. In addition, in the bottom panel of Figure 3b we show a snapshot of the propagation of the *E*-field at λ = 725 nm (resonance wavelength) that clearly shows how the energy is strongly confined in the gap exciting the (1,0) mode of the NPoM cavity.^28^

Regarding the backscattered signal coupled to the input waveguide, we needed to simulate an emitting source that imitates a Raman emitter. To this end, we considered a point-like dipolar source polarized in the *y*-direction placed in the middle of the SAM. The quantification of the coupling process is done by using the β-factor, which is defined as β = *P*_TM_/*P*_Raman_, where *P*_TM_ represents the backscattered optical power that is coupled into the fundamental TM guided mode at the input waveguide, and *P*_Raman_ is the total power emitted by the point-like source, which acts as a Raman center. In Figure 3c, we show the calculated β factor when assuming that Raman centers are simultaneously excited in the gap below both NPs. It can be seen that efficiencies of over 5% can be achieved over a broad bandwidth, reaching about 8% at some wavelengths. To illustrate how the scattered Raman signal is collected by the waveguide, in the right-side panel of the Figure 3c a snapshot is shown of the vertical electric field at λ = 750 nm when a dipole is inserted in the NPoM gap, showing that the TM mode propagates along the silicon nitride waveguide.

The integrated structure was fabricated by combining top-down and bottom-up techniques. The dielectric waveguides were defined using electron beam lithography and reactive ion etching on a SiN wafer and the Au patch was defined after using metal evaporation and lift-off (see the fabrications details in Supporting Information section SI.7). A scanning electron microscope (SEM) image of the characterized structure is shown in Figure 4b, where it can be clearly seen that two NPs are stuck on the Au patch between the waveguides. Experimental Raman measurement of the on-chip NPoM structure was conducted with a 785 nm continuous-wave Raman pump laser (13 mW power) coupled into one of the integrated waveguides using a 10× microscope objective with a numerical aperture (NA) of 0.3. The NPoM enhances the Raman interaction within its nanostructure gap, scattering a portion of the Raman signal back along the same input waveguide which is finally retro-reflected towards the objective, allowing for optimal signal collection (Figure 4a).^29^ Subsequently, the signal is directed to a 50/50 beam splitter and then filtered through a combination of bandpass and notch filters. Finally, the filtered backscattered Raman signal is conveyed to the spectrometer, where a high-sensitivity CCD (charge-coupled device) camera captures the spectra of the generated Raman light (additional information in Supporting Information section SI.8). The chip is imaged from above to capture upscattered laser light.

In Figure 4d, we show the experimentally detected Raman spectrum. For practical applications, the stability of the molecule is an important factor. To measure the stability of the molecule, we took multiple spectra at successive times, exposing the molecule to the laser over longer times (Figure 4d). The vibrational properties were stable and did not disappear under this irradiation. This allows easier molecular identification and results with good reproducibility.

Representative Raman peaks and assignment to vibrational modes are summarized in Table 1 (based on the calculated vibrational frequencies from ref (19) and experimental results of our work in Figures 2 and 4). The C–S stretching vibration gives a band in Raman in the region 750–570 cm^–l^ (peak 1).^30^ Peak no. 2 corresponds to the N–H out of plane bending vibration,^30^ and peak no. 3 corresponds to the S–H bending vibration.^21^ These three peaks are not visible in the NPoM configuration due to the low signal-to-noise ratio of this type of Raman measurements. Typically, 5-A-2MBI vibrations around 1200 cm^–1^ correspond to ν(N–C) + β(H–C–C) modes (peak no. 4), those at 1289 cm^–1^ correspond to ν(C–H) modes (peak no. 5), and those at 1338–1301 cm^–1^ correspond to ν(N–C) (peak no. 6).^18^ The C–H in plane bending mode can be identified in the range of 1370–1322 cm^–1^ assigned to peak no. 7. ν(N–C) + ν(C–C) + β(H–N–C) vibrational modes (peak no. 8) can be noted for all cases. In the case of the guided configuration, this is a broad peak that is overlapped to peak no. 7. β(N–C) + β(H–N–C) mode can be found in all cases (peak no. 9), while peak no. 10 assigned to the ν(C–C) + ν(N–C) + β(H–C–C) mode is only detectable in bulk measurement. Skeletal vibrations, involving C–C stretching modes within the ring, around 1531 cm^–1^ (peak no. 11) can be identified in the Raman response for all experimental measurements. The primary amine band is a broad band in the range of 1580–1650 cm^–1^ with a weak Raman signal, this has been assigned to peak no. 12.^30^ Moreover, this band is very close to C–C stretching mode around 1600 cm^–1^ (peak number 13) that can be overlapped with the primary amines bands as discussed in ref (30). Several factors can explain some of the differences in the Raman spectra between the SERS configurations and Bulk. NPoM and guided Raman spectra (SERS) show that the molecules are oriented in the nanogaps and that the optical field is always perpendicular to the metal facets, so there are specific selection rules for Raman transitions. Some resonances might be less strong or absent in BULK where the field and molecules are at random orientations, as was found for the case of the amine band. We note as well that bringing the molecules so close to the Au surface and also confined in this nanogap geometry can shift and change the Raman cross section of modes significantly. Currently this is hard to capture with DFT that does not include screening by the Au metal properly. Finally, the close confinement of the molecules changes their local environment, leading to broadening of the lines (also from interactions with the Au), which are clearly seen in SERS (NPoM and guided structures) versus BULK.

## Conclusions

This work presents a comprehensive method for functionalizing Au surfaces with 5-amino-2-mercaptobenzimidazole (5-A-2MBI) self-assembled monolayers (SAMs) as well as an experimental demonstration of the integration of NPoM cavities on a silicon-based photonic chip. The successful integration is verified in our experiments throughout the successful recording of Raman spectra when both the excitation and collection of the scattering are carried out via integrated waveguides.

First, we established the surface coverage, homogeneity, and 5-A-2MBI SAM stability confirmed through changes in wetting behavior, AFM topography images, and long-term Raman measurements. This SAM’s functionalization facilitated the assembly of citrate-capped gold nanoparticles (Au-NPs) to create NPoM cavities for enhancing Raman scattering. Leveraging the stability and compatibility of the 5-A-2MBI SAM with gold surfaces, we established a reliable functionalization route for gold surfaces with potential applications in catalysis, sensing, and nanotechnology. This study underscores the effectiveness and versatility of the 5-A-2MBI SAM for Au surface functionalization, offering insights into nanoscale interface design. Controlled Au-NP assembly opens possibilities for advanced materials and devices, particularly in molecular optomechanics.

Second, we provide experimental evidence that NPoM cavities can be assembled in a photonic integrated circuit so that the injection of the optical pump and the collection of the Raman scattering can be efficiently done via dielectric waveguides. To do so, we mixed standard lithographic techniques to build the waveguides and the metallic patch with a novel transfer procedure to place Au NPs on top of the path once this had been functionalized. Our results thus pave the way toward the realization of functionalities that make use of the extreme light–matter interaction taking place in NPoM cavities for applications, such as SERS or optomechanical frequency conversion, onto silicon-based photonic integrated circuits.

## Supporting Information Available

5-A-2MBI molecule and its functionalization; advancing–receding water contact angle (WCA) measurements; AFM imaging; drop-casting Au-NP deposition; NP positioning; Raman spectroscopy; waveguide-driven Raman spectroscopy; fabrication of the photonic chip; numerical simulations (PDF)

## Abbreviations

5-A-2MBI: 5-Amino-2-mercaptobenzimidazole
IR: Infrared
NP: Nanoparticle
NPoM: Nanoparticle on mirror
SAM: Self-assembled monolayer
SERS: Surface enhanced Raman spectroscopy
AFM: Atomic force microscopy
WCA: Water contact angle

## References

[ref1] Wu B.; Zheng N. Surface and Interface Control of Noble Metal Nanocrystals for Catalytic and Electrocatalytic Applications. Nano Today 2013, 8 (2), 168–197. 10.1016/j.nantod.2013.02.006.

[ref2] Lane L. A.; Qian X.; Nie S. SERS Nanoparticles in Medicine: From Label-Free Detection to Spectroscopic Tagging. Chem. Rev. 2015, 115 (19), 10489–10529. 10.1021/acs.chemrev.5b00265. 26313254

[ref3] Dugay J.; Giménez-Marqués M.; Kozlova T.; Zandbergen H. W.; Coronado E.; van der Zant H. S. J. Spin Switching in Electronic Devices Based on 2D Assemblies of Spin-Crossover Nanoparticles. Adv. Mater. 2015, 27 (7), 1288–1293. 10.1002/adma.201404441. 25556348

[ref4] Schumacher M.; Jimenez de Aberasturi D.; Merkl J.; Scarabelli L.; Lenzi E.; Henriksen-Lacey M.; Liz-Marzán L. M.; Weller H. Robust Encapsulation of Biocompatible Gold Nanosphere Assemblies for Bioimaging via Surface Enhanced Raman Scattering. Adv. Opt. Mater. 2022, 10 (14), 2102635 10.1002/adom.202102635.

[ref5] Ulman A. Formation and Structure of Self-Assembled Monolayers. Chem. Rev. 1996, 96 (4), 1533–1554. 10.1021/cr9502357. 11848802

[ref6] Love J. C.; Estroff L. A.; Kriebel J. K.; Nuzzo R. G.; Whitesides G. M. Self-Assembled Monolayers of Thiolates on Metals as a Form of Nanotechnology. Chem. Rev. 2005, 105 (4), 1103–1170. 10.1021/cr0300789. 15826011

[ref7] Hostetler M. J.; Templeton A. C.; Murray R. W. Dynamics of Place-Exchange Reactions on Monolayer-Protected Gold Cluster Molecules. Langmuir 1999, 15 (11), 3782–3789. 10.1021/la981598f.

[ref8] Gramotnev D. K.; Bozhevolnyi S. I. Plasmonics beyond the Diffraction Limit. Nat. Photonics 2010, 4 (2), 83–91. 10.1038/nphoton.2009.282.

[ref9] Rodríguez-Lorenzo L.; Álvarez-Puebla R. A.; De Abajo F. J. G.; Liz-Marzán L. M. Surface Enhanced Raman Scattering Using Star-Shaped Gold Colloidal Nanoparticles. J. Phys. Chem. C 2010, 114 (16), 7336–7340. 10.1021/jp909253w.

[ref10] Aslan K.; Gryczynski I.; Malicka J.; Matveeva E.; Lakowicz J. R.; Geddes C. D. Metal-Enhanced Fluorescence: An Emerging Tool in Biotechnology. Curr Opin Biotechnol 2005, 16 (1), 55–62. 10.1016/j.copbio.2005.01.001. 15722016 PMC6853068

[ref11] Langer J.; Jimenez de Aberasturi D.; Aizpurua J.; Alvarez-Puebla R. A.; Auguié B.; Baumberg J. J.; Bazan G. C.; Bell S. E. J.; Boisen A.; Brolo A. G.; Choo J.; et al. Present and Future of Surface-Enhanced Raman Scattering. ACS Nano 2020, 14 (1), 28–117. 10.1021/acsnano.9b04224. 31478375 PMC6990571

[ref12] Nie S.; Emory S. R. Probing Single Molecules and Single Nanoparticles by Surface-Enhanced Raman Scattering. Science (1979) 1997, 275 (5303), 1102–1106. 10.1126/science.275.5303.1102. 9027306

[ref13] Benz F.; Schmidt M. K.; Dreismann A.; Chikkaraddy R.; Zhang Y.; Demetriadou A.; Carnegie C.; Ohadi H.; De Nijs B.; Esteban R.; Aizpurua J.; Baumberg J. J. Single-Molecule Optomechanics in “Picocavities.”. Science (1979) 2016, 354 (6313), 726–729. 10.1126/science.aah5243. 27846600

[ref14] Baumberg J. J.; Aizpurua J.; Mikkelsen M. H.; Smith D. R. Extreme Nanophotonics from Ultrathin Metallic Gaps. Nat Mater 2019, 18 (7), 668–678. 10.1038/s41563-019-0290-y. 30936482

[ref15] Redolat J.; Camarena-Pérez M.; Griol A.; Kovylina M.; Xomalis A.; Baumberg J. J.; Martínez A.; Pinilla-Cienfuegos E. Accurate Transfer of Individual Nanoparticles onto Single Photonic Nanostructures. ACS Appl Mater Interfaces 2023, 15 (2), 3558–3565. 10.1021/acsami.2c13633. 36538469 PMC9869328

[ref16] Xomalis A.; Zheng X.; Chikkaraddy R.; Koczor-Benda Z.; Miele E.; Rosta E.; Vandenbosch G. A. E.; Martínez A.; Baumberg J. J. Detecting Mid-Infrared Light by Molecular Frequency Upconversion in Dual-Wavelength Nanoantennas. Science (1979) 2021, 374 (6572), 1268–1271. 10.1126/science.abk2593. 34855505

[ref17] Chen W.; Roelli P.; Hu H.; Verlekar S.; Amirtharaj S. P.; Barreda A. I.; Kippenberg T. J.; Kovylina M.; Verhagen E.; Martínez A.; Galland C. Continuous-Wave Frequency Upconversion with a Molecular Optomechanical Nanocavity. Science (1979) 2021, 374 (6572), 1264–1267. 10.1126/science.abk3106. 34855500

[ref18] Chen Y.; Yang J.; Li Z.; Li R.; Ruan W.; Zhuang Z.; Zhao B. Experimental and Density Functional Theory Study of Raman and SERS Spectra of 5-Amino-2-Mercaptobenzimidazole. Spectrochim Acta A Mol Biomol Spectrosc 2016, 153, 344–348. 10.1016/j.saa.2015.08.039. 26335062

[ref19] Koczor-Benda Z.; Roelli P.; Galland C.; Rosta E. Molecular Vibration Explorer: An Online Database and Toolbox for Surface-Enhanced Frequency Conversion and Infrared and Raman Spectroscopy. J Phys Chem A 2022, 126 (28), 4657–4663. 10.1021/acs.jpca.2c03700. 35792893 PMC9310003

[ref20] Leone G.; Consumi M.; Lamponi S.; Magnani A. Combination of Static Time of Flight Secondary Ion Mass Spectrometry and Infrared Reflection-Adsorption Spectroscopy for the Characterisation of a Four Steps Built-up Carbohydrate Array. Appl Surf Sci 2012, 258 (17), 6302–6315. 10.1016/j.apsusc.2012.03.027.

[ref21] Bazylewski P.; Divigalpitiya R.; Fanchini G. In Situ Raman Spectroscopy Distinguishes between Reversible and Irreversible Thiol Modifications in L-Cysteine. RSC Adv 2017, 7 (5), 2964–2970. 10.1039/C6RA25879D.

[ref22] Coronado E.; Forment-Aliaga A.; Pinilla-Cienfuegos E.; Tatay S.; Catala L.; Plaza J. A. Nanopatterning of Anionic Nanoparticles Based on Magnetic Prussian-Blue Analogues. Adv Funct Mater 2012, 22 (17), 3625–3633. 10.1002/adfm.201200067.

[ref23] Ettabib M. A.; Marti A.; Liu Z.; Bowden B. M.; Zervas M. N.; Bartlett P. N.; Wilkinson J. S. Waveguide Enhanced Raman Spectroscopy for Biosensing: A Review. ACS Sens 2021, 6 (6), 2025–2045. 10.1021/acssensors.1c00366. 34114813

[ref24] Peyskens F.; Dhakal A.; Van Dorpe P.; Le Thomas N.; Baets R. Surface Enhanced Raman Spectroscopy Using a Single Mode Nanophotonic-Plasmonic Platform. ACS Photonics 2016, 3 (1), 102–108. 10.1021/acsphotonics.5b00487.

[ref25] Losada J.; Raza A.; Clemmen S.; Serrano A.; Griol A.; Baets R.; Martinez A. SERS Detection via Individual Bowtie Nanoantennas Integrated in Si _3_ N _4_ Waveguides. IEEE Journal of Selected Topics in Quantum Electronics 2019, 25 (3), 1–6. 10.1109/JSTQE.2019.2896200.

[ref26] Vázquez-Lozano J. E.; Baumberg J. J.; Martínez A. Enhanced Excitation and Readout of Plasmonic Cavity Modes in NPoM via SiN Waveguides for On-Chip SERS. Opt Express 2022, 30 (3), 4553 10.1364/OE.446895. 35209689

[ref27] Dhakal A.; Wuytens P.; Raza A.; Le Thomas N.; Baets R. Silicon Nitride Background in Nanophotonic Waveguide Enhanced Raman Spectroscopy. Materials 2017, 10 (2), 140 10.3390/ma10020140. 28772499 PMC5459205

[ref28] Kongsuwan N.; Demetriadou A.; Horton M.; Chikkaraddy R.; Baumberg J. J.; Hess O. Plasmonic Nanocavity Modes: From near-Field to Far-Field Radiation. ACS Photonics 2020, 7 (2), 463–471. 10.1021/acsphotonics.9b01445.

[ref29] Miele E.; Dose W. M.; Manyakin I.; Frosz M. H.; Ruff Z.; De Volder M. F. L.; Grey C. P.; Baumberg J. J.; Euser T. G. Hollow-Core Optical Fibre Sensors for Operando Raman Spectroscopy Investigation of Li-Ion Battery Liquid Electrolytes. Nat Commun 2022, 13 (1), 1651 10.1038/s41467-022-29330-4. 35347137 PMC8960792

[ref30] Socrates G. Infrared and Raman Characteristic Group Frequencies: Tables and Charts, 3rd ed.; Wiley: Chichester, U.K, 2001.

