# Supplementary material for: Synthesis and Raman Detection of 5-Amino-2-mercaptobenzimidazole Self-Assembled Monolayers in Nanoparticle-on-a-Mirror Plasmonic Cavity Driven by Dielectric Waveguides
Source: Nano Lett. 2024 Mar 14;24(12):3670–7. doi: 10.1021/acs.nanolett.3c04932 (PMC10979432; doi:10.1021/acs.nanolett.3c04932)
Supplement: Supplementary file 1 — nl3c04932_si_001.pdf [file nl3c04932_si_001.pdf]

Supporting Information.

Synthesis and Raman detection of 5-amino-2-mercaptobenzimidazole self-assembled monolayers in nanoparticle-on-a-mirror plasmonic cavity driven by dielectric waveguides

Javier Redolat[1‡], María Camarena-Pérez[1], Amadeu Griol[1], Miguel Sinusia Lozano[1], Maria Isabel Gómez-Gómez[1], J. Enrique Vázquez-Lozano[2], Ermanno Miele[3], Jeremy J. Baumberg[3], Alejandro Martínez, [1] and Elena Pinilla-Cienfuegos [1*]*

1. Nanophotonics Technology Center, Universitat Politècnica de València, Valencia E46022, Spain.

2. Department of Electrical, Electronic and Communications Engineering, Institute of Smart Cities (ISC), Universidad Pública de Navarra (UPNA), 31006 Pamplona, Spain

3. NanoPhotonics Centre, Cavendish Laboratory, Department of Physics, University of Cambridge, Cambridge, CB3 0HE, United Kingdom.

SI.1: Advancing-Receding Water Contact Angle (WCA) measurements

Advancing and receding water contact angle measurements were performed using the "add and remove volume" method in a Ramé-hart Model 90 Standard Goniometer with Dropimage Standard software equipped with an automated dispensing system. This device includes software and an LED illuminator, a 3-axis levelling stage, a digital camera, a microsyringe fixture and assembly for manual dispensing. The system is improved with an automated dispensing system and manual tilting base.

The advancing and receding contact angles of functionalized and non-functionalized samples were performed using the needle-in-sessile-drop method at room temperature without humidity control. Initially, a deionized water droplet of approximately 1mL was dispensed onto the sample surface using a motorized micro syringe. Subsequently, water was added to the droplet in tiny increments (1μL) at each step, and the contact angle was recorded. Water was added to the droplet to determine the receding contact angle until

the minimum angle was observed. This advancing-receding cycle was repeated three times. The mean values of the maximum (minimum) angles from the remaining four cycles of the advancing (receding) contact angles were calculated.

Three samples were used to measure the differences between a 5-A-2MBI functionalized and a non-functionalized gold surface. Figure S.1a shows the results of the WCA measurements, which correspond to the WCA of a non-functionalized Au sample, bare Au substrate immersed for 12h in Absolute Ethanol and finally a Au-5-A-2MBI functionalized sample. Different WCA values were obtained for each case, probing that the CA method is useful for probing the surface functionalization: 76° for non-functionalized gold, 62° for non-functionalized gold + Ethanol, and 54° for functionalized gold.

As expected, 5-A-2MBI SAM provides more hydrophilic surfaces when compared with bare Au surface, attributed to the presence of amines and other N groups.

SI.2: Atomic Force Microscopy (AFM) imaging

An Alpha300 RA (Raman-AFM) instrument from WITec was employed for the AFM sample characterization. All measurements were performed in AC mode. Sharp silicon

probes without coating (K ~ 42 N/m, f_0 ~ 320 kHz) were purchased from PPP-NCH (Nanosensors). All AFM images were processed with WSxM software from Nanotec Electrónica S.L.[1]

Figure S1b displays an AFM topography image of an Au-5-A-2MBI functionalized surface, revealing a flat and homogeneous appearance (RMS = 0,75) without clusters or islands. Additionally, in Figure S1(b,c), an Au-5-A-2MBI functionalized sample is depicted after 60 nm diameter gold nanoparticles (Au-NPs) drop-casting deposition. The Au-NPs are randomly dispersed onto the Au-5-A-2MBI functionalized surface (RMS = 1).

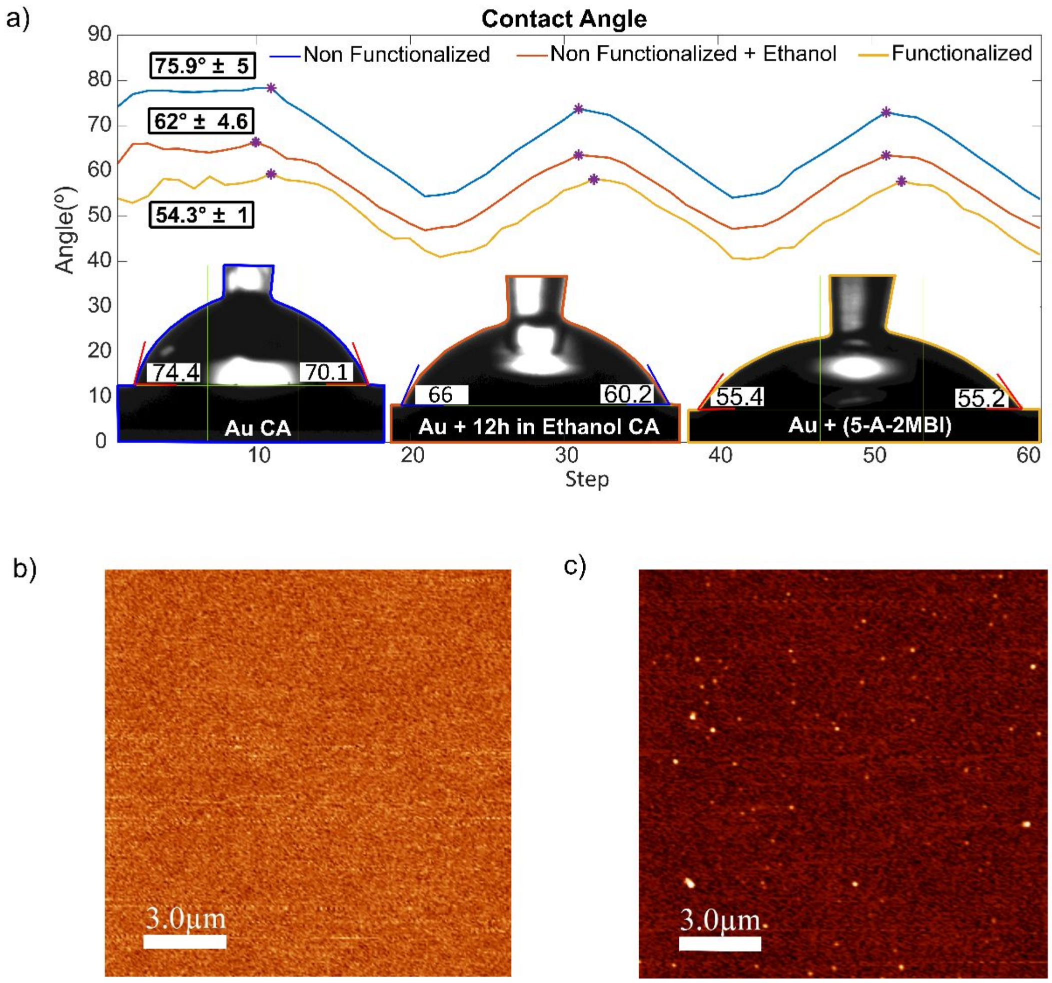

Figure S1. a) The blue curve corresponds to the non-functionalized Au sample, while the orange curve represents the CA of bare gold immersed in Ethanol for 12h, and the red one represents the WCA measurements for the Au-5-A-2MBI functionalized sample. Insets: Optical images of a single water droplet for each case. b) AFM topography image of an Au-5-A-2MBI functionalized sample (15 x 15 µm^2). c) AFM topography image after performing drop casting Au-NPs deposition (15 x 15 µm^2).

SI.3: Drop casting Au-NP deposition

The drop casting was performed by delivering an 8 μL drop of 60 nm Au-NP solution onto the 5-A-2MBI functionalized Au sample, left for 5 minutes and then rinsed with Mili-Q water. Finally, the substrate was dried under the N_2 stream. Water suspension of spheric Citrate-capped 60 nm Au-NPs were purchased from Nanopartz™. The substrate was protonated to improve the NP's delivery. Protonation is a technique that harnesses the property of certain molecules to become positively charged. In this case, the 5-A-2MBI molecule has an amine group $-NH_2$, which can eventually become $-NH_3^+$ (Figure S.2a). This method consists of immersing the sample in a strong acid solution as hydrochloric acid (HCl). Consequently, the surface will acquire a positive charge, making it more efficient in attracting negatively charged Au-NPs. To quantify the improvement in sample protonation for Au-NPs deposition, we conducted large-area optical dark-field imaging at 50x magnification (From Figure S.2b to i). Figure S.2b and Figure S.2c display dark-field optical images taken before and after sample protonation, followed by drop casting of 60 nm and 150 nm diameter Au-NPs, respectively. We can see an increase in the density of Au-NPs for both NP sizes. Also, a better distribution of single NPs on the gold surface can be seen, especially for NPs of 60 nm in a functionalized Au surface. In percentages, considering the bare gold, the density increases by approximately 600 %

after protonation and 800% after functionalized surface protonation for 60nm Au-NPs. For 150 nm Au-NPs, the increment of the NP numbers for a bare gold surface is around 42 %, and there is an increase of approximately 300 % in the density of 150 nm Au-NPs for protonated functionalized surfaces.

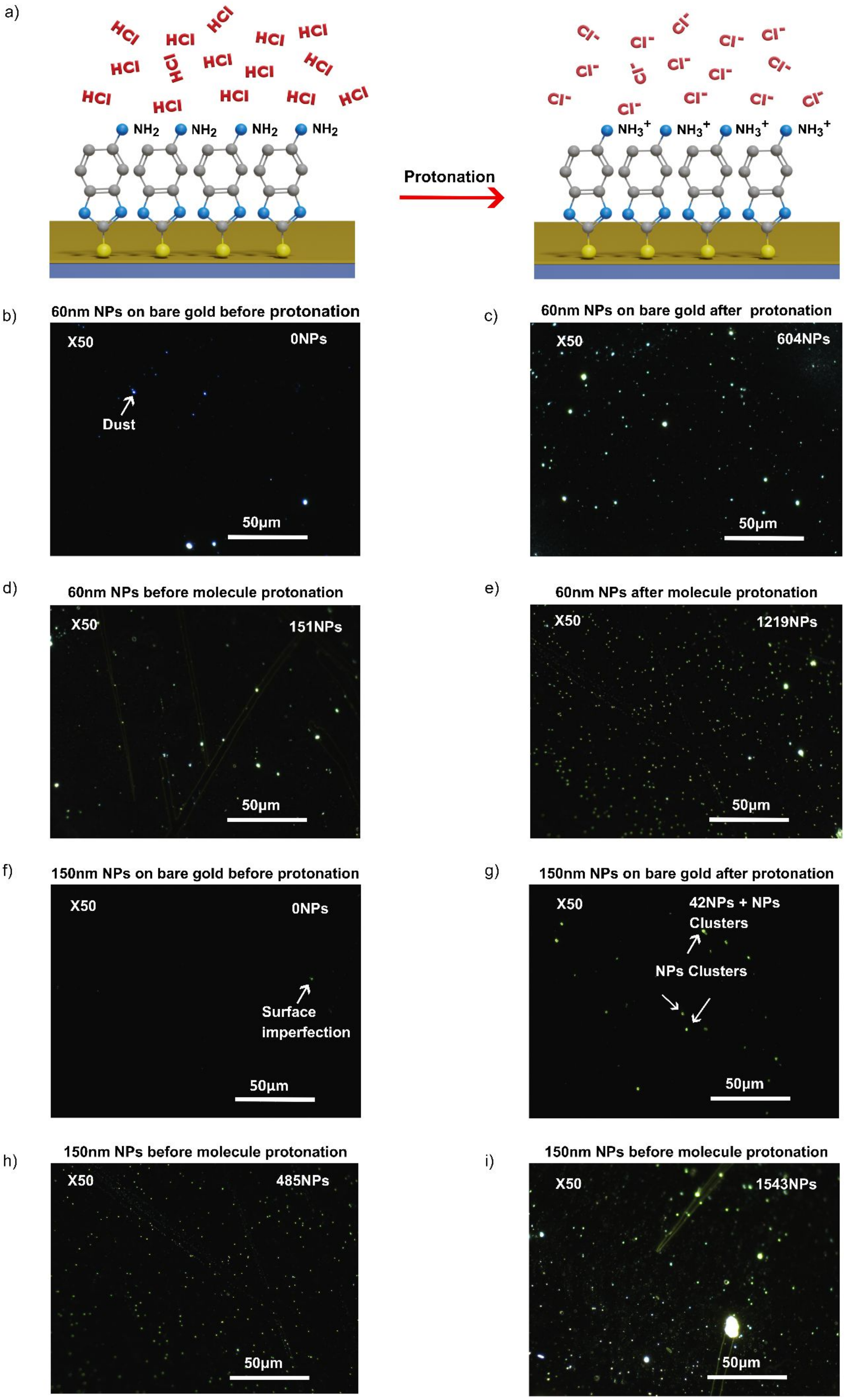
a)
HCl
NH2
Protonation
Cl-
NH3+
b)
60nm NPs on bare gold before protonation
X50
0NPs
Dust
50µm
c)
60nm NPs on bare gold after protonation
X50
604NPs
50µm
d)
60nm NPs before molecule protonation
X50
151NPs
50µm
e)
60nm NPs after molecule protonation
X50
1219NPs
50µm
f)
150nm NPs on bare gold before protonation
X50
0NPs
Surface imperfection
50µm
g)
150nm NPs on bare gold after protonation
X50
42NPs + NPs Clusters
NPs Clusters
50µm
h)
150nm NPs before molecule protonation
X50
485NPs
50µm
i)
150nm NPs before molecule protonation
X50
1543NPs
50µm

Figure S 2. a) Schematic representation of the surface protonation method using HCl as a strong acid. b) A dark field optical image at 50x magnification shows a drop-casting of 60 nm Au-NPs bare gold before protonation. c) A dark field optical image at 50x magnification shows a drop-casting of 60 nm Au-NPs bare gold after protonation. d) A dark field optical image at 50x magnification shows a drop-casting of 60 nm Au-NPs functionalized sample before protonation e) A dark field optical image at 50x magnification shows a drop-casting of 60 nm Au-NPs functionalized sample after protonation. f) A dark field optical image at 50x magnification shows a drop-casting of 1500 nm Au-NPs bare gold after protonation. g) A dark field optical image at 50x magnification shows a drop-casting of 150 nm Au-NPs functionalized sample before protonation. h) A dark field optical image at 50x magnification shows a drop-casting of 150 nm Au-NPs functionalized sample before protonation. i) A dark field optical image at 50x magnification shows a drop-casting of 150 nm Au-NPs functionalized sample after protonation.

SI.4: Raman spectroscopy

Spectrometer alpha300 RA (Raman-AFM) from WITec provides information about the AntiStokes Raman scattering in the 80 - 3000 cm^{-1} range, with monochromatic 532 nm

(green) and 633 nm (red) laser illumination and 100x objective. Raman measurements were performed at 532%nm (green) excitation, P = 7 mW power, grating G = 600 l/mm and objective 100x. The Raman images were scanned at 300%×%300 points, with 0.035%s integration time at each point.

SI.5: NP positioning

Spherical citrate-capped 150 nm Au-NPs were deterministically positioned on top of plasmonic nanocavities consisting of a 650 x 650 nm squared Au patch lithographed at the intersection of two orthogonal 600 x 220 nm Si_3N_4 waveguides. After functionalization of the Au patch, the transfer of 150 nm Au-NPs was performed using a micro-contact printing technique developed by our group, allowing the controlled positioning of individual Au NPs with sub-micron accuracy.[2] Water suspensions of spheric Citrate-capped 150 nm Au-NPs were purchased from Nanopartz™.

SI.6: Numerical simulations

Simulations were conducted based on the geometries described in the main text. The simulations were performed according to the main text. Figure S3a shows the structure with the port positions, which are essential for the whole study. The results were obtained by considering two separate modes: excitation and collection. On the one hand, for the excitation, a TM coupling mode (Figure S3b) had to be considered so that the electric field was vertically oriented, allowing interaction with the 5-A-2MBI SAM. Additionally, we assumed that Port 1 is the input port where the electromagnetic field is coupled to the waveguide. The enhanced electric field was measured by placing probes in the middle of the gap between the nanoparticle and the gold surface (Figure S3c).

On the other hand, to assess the theoretical collection of the emitted signal, we considered a point dipole exactly in the same location where the probes were positioned, in the middle of the SAM below the NP (Figure S3d). The signal is emitted by the dipole, coupled to the waveguide, and directed towards Port 1, whose S-parameters indicate the intensity. Simulations were conducted with and without a metallic structure to study the SERS effect of the structure.

The mesh played an important role in optimizing the calculation time. For this purpose, a hexahedral mesh was considered, paying particular attention to critical areas such as the

gap, where a more precise mesh was considered. Additionally, the waveguide is made of silicon nitride (n=2), and a refractive index of n=1.8 was considered for the SAM,[3] n=1.45 for the SiO_2 structure on which the gold patch is positioned. For all gold structures, the specifications of Johnson and Christy from 1972 were selected.[4]

.

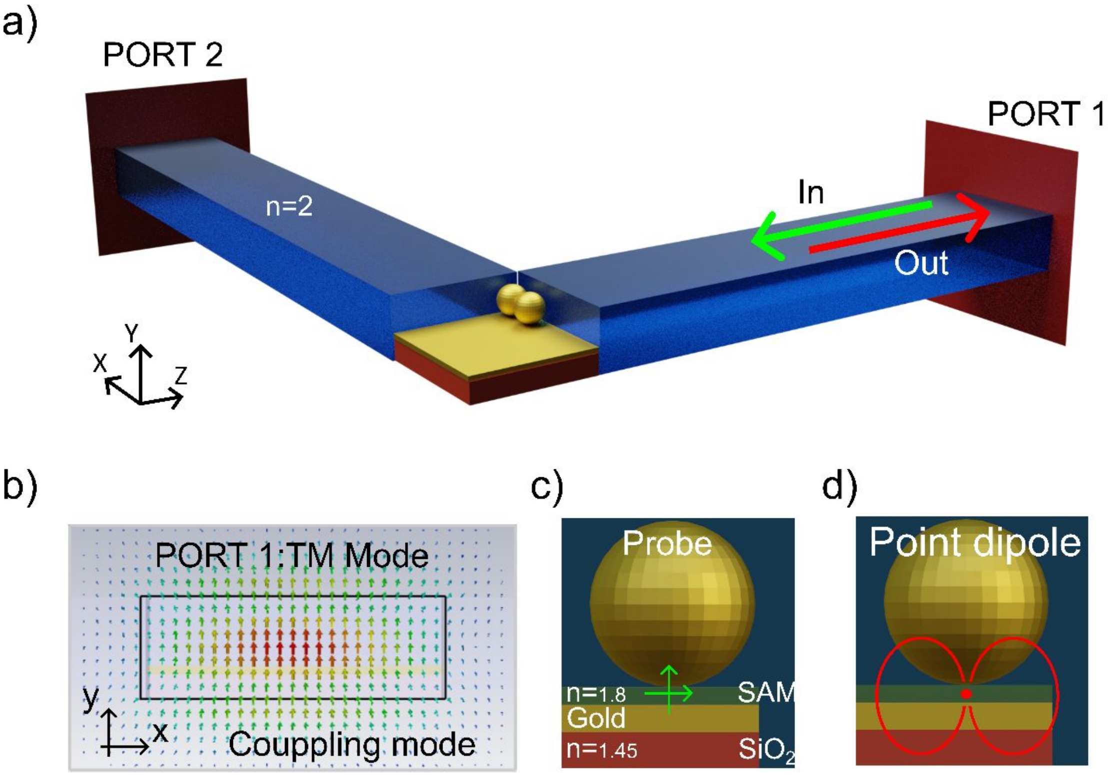

Figure S 3. a) Scheme of the complete simulated structure with the specifications of the Ports positions. b) EM-guided excitation mode, where the arrows show the orientation of the Electric field. c) Scheme of the position of the probes, where the Electric field was measured. d) Scheme of the location of the like-point dipole to evaluate the emission and β-factor.

SI.7: Fabrication of the photonic chip

The waveguide structures were fabricated on standard silicon nitride samples with a thickness of 300 nm and a buried oxide layer thickness of 3.27 μm. The fabrication is based on an electron-beam (e-beam) direct-writing process performed on a coated 300 nm negative (Man-2403) resist film. The mentioned e-beam exposure, performed with a Raith150 tool, was optimized to reach the required dimensions, employing an acceleration voltage of 20 KeV and an aperture size of 30 um. After developing, the resist patterns were transferred into the Silicon nitride employing an optimized Inductively Coupled Plasma-Reactive Ion Etching process with fluoride gases. A second lithography e-beam process, in this case using positive resist PMMA, was carried out to fabricate the metal patch prior to an evaporation process of 40 nm of gold and a lift-off process employing MNP as solvent.

SI.8: Waveguide-driven Raman spectroscopy

Spectrometer alpha300 RA (Raman-AFM) from WITec provides information about the AntiStokes Raman scattering in the 80 - 3000 cm−1 range, with monochromatic 532 nm (green) and 633 nm (red) laser illumination and 100x objective. Raman measurements were performed at 532%nm (green) excitation, a power of P = 7 mW, a grating G = 600

l/mm and a 100x objective. The Raman images were scanned at 300%×%300 points, with 0.035%s integration time at each point.

A 785 nm diode laser was coupled using an 40x objective into the mounted photonic chip, with an imaging camera collecting scattered light from above to a certain when coupling into the waveguide was achieved. Before reaching the waveguide, the laser passed through a 50% beam splitter, so returning light from the waveguide could be collected and focused into a monochromator and CCD (Andor) for measuring Raman spectra. These were calibrated using the signal from the Si surface.

REFERENCES

(1) Horcas, I.; Fernández, R.; Gómez-Rodríguez, J. M.; Colchero, J.; Gómez-Herrero, J.; Baro, A. M. WSXM: A Software for Scanning Probe Microscopy and a Tool for Nanotechnology. *Review of Scientific Instruments* **2007**, *78* (1), 39.

(2) Redolat, J.; Camarena-Pérez, M.; Griol, A.; Kovylina, M.; Xomalis, A.; Baumberg, J. J.; Martínez, A.; Pinilla-Cienfuegos, E. Accurate Transfer of Individual Nanoparticles onto Single Photonic Nanostructures. *ACS Appl Mater Interfaces* **2023**, *15* (2), 3558–3565.

(3) Https://Www.Alfa-Chemical.Com/Organic-Chemistry/Chemical-Reagents/Cas-2818-66-8-5-Amino-2-Mercaptobenzimidazole.Html.

(4) Koelling, D.; Freeman, A.; Mueller, F.; Johnson, P. B.; Christy, R. W. PHYSICA L RE VIEW B VOLUME. *Phys. Rev. Letters* **1963**, *11*, 3093.
